# Rapid and Low-Cost CRP Measurement by Integrating a Paper-Based Microfluidic Immunoassay with Smartphone (CRP-Chip)

**DOI:** 10.3390/s17040684

**Published:** 2017-03-26

**Authors:** Meili Dong, Jiandong Wu, Zimin Ma, Hagit Peretz-Soroka, Michael Zhang, Paul Komenda, Navdeep Tangri, Yong Liu, Claudio Rigatto, Francis Lin

**Affiliations:** 1 Institute of Applied Technology, Hefei Institutes of Physical Science, Chinese Academy of Sciences, Hefei 230031, China; dongmeili@aiofm.ac.cn; 2 Department of Physics and Astronomy, University of Manitoba, Winnipeg, MB R3T 2N2, Canada; wujd@physics.umanitoba.ca (J.W.); zimin6687@gmail.com (Z.M.); hagit.peretz@gmail.com (H.P.-S.); 3 Seven Oaks General Hospital, Winnipeg, MB R2V 3M3, Canada; GZHANG@wrha.mb.ca (M.Z.); pkomenda@sogh.mb.ca (P.K.); ntangri@sogh.mb.ca (N.T.); 4 Department of Biosystems Engineering, University of Manitoba, Winnipeg, MB R3T 2N2, Canada; 5 Department of Immunology, University of Manitoba, Winnipeg, MB R3T 2N2, Canada; 6 Department of Biological Sciences, University of Manitoba, Winnipeg, MB R3T 2N2, Canada

**Keywords:** C-reactive protein, protein biomarkers, chronic disease diagnosis, microfluidic device, smartphone, low-cost

## Abstract

Traditional diagnostic tests for chronic diseases are expensive and require a specialized laboratory, therefore limiting their use for point-of-care (PoC) testing. To address this gap, we developed a method for rapid and low-cost C-reactive protein (CRP) detection from blood by integrating a paper-based microfluidic immunoassay with a smartphone (CRP-Chip). We chose CRP for this initial development because it is a strong biomarker of prognosis in chronic heart and kidney disease. The microfluidic immunoassay is realized by lateral flow and gold nanoparticle-based colorimetric detection of the target protein. The test image signal is acquired and analyzed using a commercial smartphone with an attached microlens and a 3D-printed chip–phone interface. The CRP-Chip was validated for detecting CRP in blood samples from chronic kidney disease patients and healthy subjects. The linear detection range of the CRP-Chip is up to 2 μg/mL and the detection limit is 54 ng/mL. The CRP-Chip test result yields high reproducibility and is consistent with the standard ELISA kit. A single CRP-Chip can perform the test in triplicate on a single chip within 15 min for less than 50 US cents of material cost. This CRP-Chip with attractive features of low-cost, fast test speed, and integrated easy operation with smartphones has the potential to enable future clinical PoC chronic disease diagnosis and risk stratification by parallel measurements of a panel of protein biomarkers.

## 1. Introduction

The burden of chronic major organ disease (e.g., cardiovascular, chronic kidney disease) is reaching epidemic proportions in both the developed and developing world [1,2,3]. Most chronic diseases are eminently treatable, provided patients are diagnosed early. However, as the existing therapies carry significant risks of adverse side-effects, they must be targeted only to patients with a sufficiently high risk of downstream complications, so that a net benefit of therapy is realized [4,5]. For these reasons, early diagnosis and accurate risk assessment are the cornerstones of chronic diseases management, and are both essential in preventing downstream morbidity and mortality for patients. Traditionally, conventional clinical and laboratory variables have been used to diagnose and predict risk for progression in chronic diseases. These variables have often been combined into risk prediction equations. Although many of these equations are widely used [6,7], they remain imperfect prediction tools. For this reason, there is ongoing interest in developing novel biomarkers of cardiac and renal disease. Several novel protein biomarkers, each reflecting underlying biological processes associated with adverse disease outcomes, have been identified and shown to improve clinical prediction [8,9,10].

Unfortunately, clinical validation and translation of newer protein-based biomarkers have been slowed by the cost and inconvenience of biomarker measurement. Enzyme-linked immunosorbent assay (ELISA), one of the standard protein detection methods [11,12], is expensive, time-consuming and requires specialized laboratory facilities and skills. It is therefore ill-suited for routine clinical testing at the point-of-care (PoC). Microfluidics-based immunoassays offer significant advantages in cost-factor, reduced sample and reagent requirement, ease-of-operation, high test throughput, and integration owing to miniaturization [13,14,15]. Various materials can be used for fabricating microfluidic devices such as polymers, thermoplastic, glass, cloth, and paper [16]. Among them, microfluidic paper-based analytical device (μPAD) offers the benefits of low-cost, easy fabrication, and self-powered fluidic transport by capillary force [17,18]. Compared to a traditional paper test strip, μPAD can control fluidic transport within hydrophilic channels defined by printed hydrophobic barriers [19]. Over recent years, μPAD has been increasingly developed for various chemical, biochemical, and biological applications [20] such as protein assays [21,22], blood separation [23], and even cell migration testing [24]. Another closely-related emerging research area is mobile sensing based on the integration of microfluidic device and smartphone, termed MS^2^ technology [25]. Various MS^2^-based immunoassays have been developed to enable PoC biomedical applications [26,27].

Here, we developed a MS^2^-based μPAD immunoassay for rapid and low-cost CRP detection from a blood sample (i.e., CRP-Chip). We chose CRP for this initial development because it is a strong biomarker of adverse outcomes in both chronic heart and kidney disease [28,29,30]. The paper-based microfluidic immunoassay was realized by lateral flow assay (LFA) and gold nanoparticle based colormetric detection of the target protein. The test image signal was acquired using a commercial smartphone with an attached microlens and a 3D-printed chip-phone interface. A smartphone app was also developed for image acquisition and data analysis of the CRP-Chip. The developed CRP-Chip was successfully validated against human CRP standard, plasma, and whole blood. The useful features of this CRP-Chip are fast testing (within 15 min), low-cost (<50 US cents of material cost per test), high reproducibility, and integration with smartphones for easy signal readout. This developed CRP-Chip provides the initial proof-of-concept of MS^2^-based μPAD for rapid and low-cost immunoassay of chronic disease related protein biomarkers. On the other hand, detection of other biomarkers based on this general method will still require independent development, characterizations, and optimization.

## 2. Materials and Methods

### 2.1. Materials and Chemicals for the CRP-Chip Test

Purified human CRP (CRP15-N-100), goat anti human CRP (CRP11-A), goat anti-mouse IgG (40121) and mouse anti-hCRP IgG gold conjugate (CRP13-GLD) were purchased from Alpha Diagnostic Intl. Inc (San Antonio, TX, USA). One wt.% DPBS aqueous solution was used as the dilution buffer for blood sample, plasma sample, and BSA. Point four wt.% BSA solution was used for blocking the nitrocellulose membrane and glass fiber. The nitrocellulose membrane (HF09004X SS), absorbent paper (CFSP 223000) and glass fiber membrane (CFDX 203000) were purchased from Millipore (Billerica, MA, USA). The plasma separation membrane (T9EXPPA00200S00A) was purchased from Vivid Pall (Port Washington, NY, USA).

### 2.2. Blood Samples

We obtained whole blood from 10 healthy control subjects and 17 patients with CKD participating in a prospective study. All samples were obtained at the Seven Oaks General Hospital for CKD patient samples (Winnipeg, MB, Canada) and Victoria General Hospital for healthy blood samples (Winnipeg, MB, Canada) under ethics protocols (protocol #: H2014:185; J2015:022) approved by the University of Manitoba (Winnipeg, MB, Canada). All human subjects were recruited with informed written consent. Plasma and serum samples separated from whole blood were stored at −80 °C until use. 

### 2.3. Paper Devices Fabrication

AutoCAD software (Autodesk) was used to design the pattern of the paper devices. The test strip consists of five components: (1) a sample pad made of plasma separation membrane; (2) a conjugate pad; (3) a nitrocellulose membrane; (4) an absorbent pad; and (5) a backing pad. A solid wax ink printer (ColorQube 8570, Xerox, Norwalk, CT, USA) was used to print the pattern on the nitrocellulose membrane (1 mm-wide flow channels defined by printed wax ink boundaries) and plasma separation membrane (a circle defined by the printed ink boundary). The printed wax pattern was briefly heated on a hotplate at 125 °C to allow melted wax ink to enter the membrane and form the hydrophobic boundaries. Round 7 mm-diameter absorbent pad and conjugate pad were made using a puncher. A desktop craft cutter (Silhouette America, Inc., Lindon, UT, USA) was used to cut the backing pad. 

The anti-CRP capture antibody (0.1 μL at 200 ng/μL) was spotted on the nitrocellulose membrane in the three test channels of the CRP-Chip as the test point (T-point). In addition, the anti-IgG (0.1 μL at 1 mg/mL) was deposited on the nitrocellulose membrane in the three test channels of the CRP-Chip after each test point as the control point (C-point). The distance between C-point and T-point is about 3 mm. The spotted membrane was dried overnight at room temperature. The dried nitrocellulose membrane was then blocked with 0.4% BSA in PBS. The conjugate pad was pretreated by 0.4% BSA in PBS and then 2 μL of the anti-CRP gold nanoparticle (AuNP) conjugated detection antibody was added to the conjugate pad. The pad was dried at room temperature before use. 

**Figure 1:**
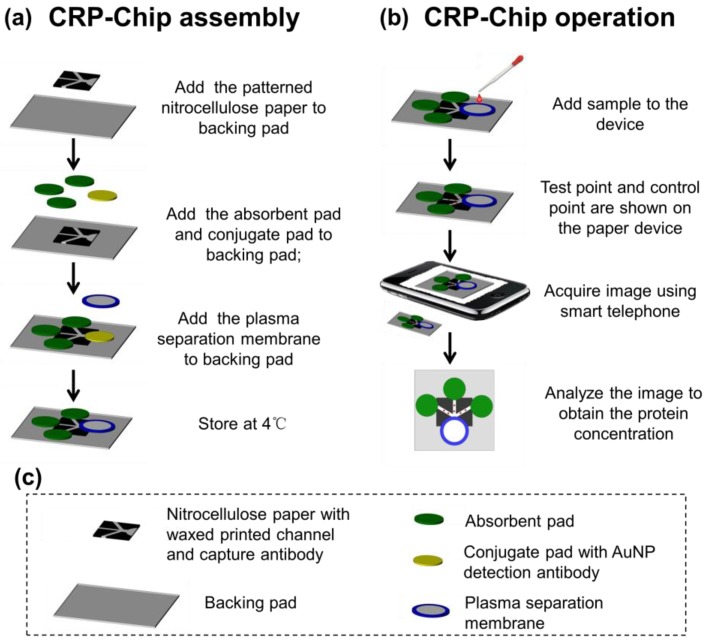
Illustration of CRP-Chip assembly (**a**) and operation (**b**). (**c**) Description of components of the CRP-Chip.

The CRP-Chip was assembled as illustrated in Figure 1a. Briefly, the nitrocellulose pad with printed flow channels and spotted test and control capture antibodies was attached to the backing pad. Then the absorbent pads were attached to the backing pad. Next, the conjugate pad with the spotted gold nanoparticle (AuNP) conjugated detection antibody pad was attached to the nitrocellulose pad with the printed sample loading area. Finally, the sample pad made of the plasma separation membrane was attached to the center of the conjugate pad. The completed CRP-Chip was used for experiment immediately or stored at 4 °C until use.

### 2.4. CRP-Chip Test Protocol

**Figure 2:**
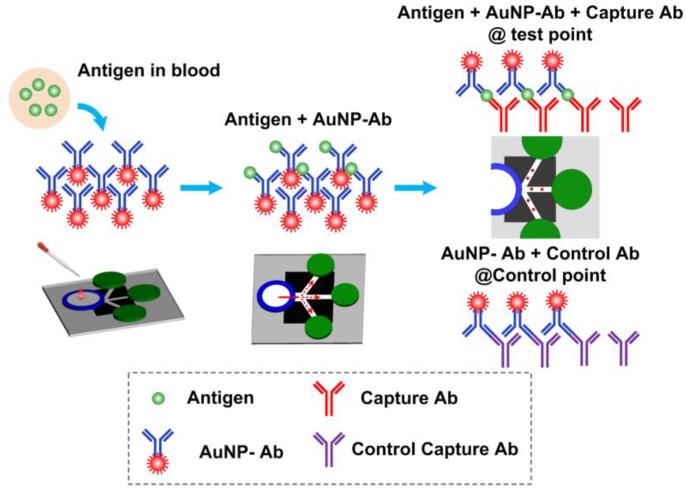
Illustration of the CRP detection principle using the CRP-Chip.

The CRP-Chip test is based on lateral flow sandwiched immunoassay in a MS^2^ format as illustrated in Figures Figure 1b and Figure 2. First, 20 μL of sample was dispensed onto the sample pad. For whole blood, plasma was isolated by the plasma isolation membrane and directed to the conjugate pad. Serum and plasma sample can be directly dispensed onto the conjugated pad without the plasma isolation membrane. The CRP in the sample bound specifically to the AuNP conjugated detection antibody. The sample containing detection antibody labeled CRP and free AuNP conjugated detection antibody flow along the printed channels on the nitrocellulose membrane through capillary action. The detection antibody labeled CRP is immobilized by the anti-CRP captured antibody at the T-point and shows a color spot. Similarly, the free AuNP-conjugated detection antibody is immobilized by the control capture antibody at the C-point and shows another color spot. Then 40 μL PBS is added to the sample pad or conjugated pad to wash the channels. After drying, the colormetric CRP test signal in the CRP-Chip is imaged using the smartphone module. The intensity of the T-point is calibrated to indicate the CRP concentration in the sample and the C-point acts as a positive control. The same batch of CRP-Chips was always used for the batch-specific linear detection range calibration based on the CRP standard, isolated blood plasma, and whole blood from healthy blood donors. 

### 2.5. Polydimethylsiloxane (PDMS) Microlens Fabrication

The PDMS base and curing agent were mixed at the ratio of 10:1 and degassed by a vacuum desiccator. A drop (~100 µL) of PDMS was dispensed onto the surface of a microscope glass slide using a hypodermic needle and syringe and baked at 70 °C for 15 min to form the PDMS substrate. Another drop (~100 µL) of PDMS was dispensed onto the PDMS substrate and flipped over the glass slide immediately. This will create a semi-spherical PDMS droplet by gravity. The PDMS droplet was baked at 70 °C for 2 h. The cured PDMS droplet is used as a microlens and can be easily attached to the smartphone camera for amplifying the image signal from the CRP-Chip test. 

### 2.6. Smartphone Imaging

**Figure 3:**
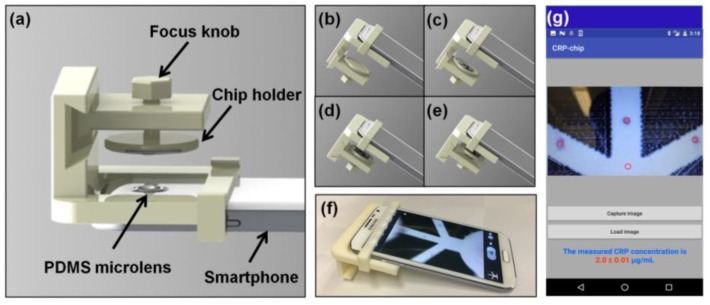
Illustration of the smartphone module for the CRP-Chip test. (**a**) The 3D printed chip-phone interface and the PDMS microlens attachment; (**b**–**e**) Smartphone imaging process: move out the chip holder (**b**); insert the CRP-Chip to the chip holder (**c**); return the chip holder and the center it to the PDMS microlens/smartphone camera (**d**); adjust the focus using the focus knob and take the image (**e**); (**f**) A picture of the real system. (**g**) Interface of the smartphone app for image acquisition and data analysis.

A commercial brand smartphone with an attached PDMS microlens was used to capture the CRP-Chip test signal (Figure 3). A chip-phone interface attachment was fabricated using a 3D printer. The interface attachment consists of a focus knob, a chip holder, and the mounting frame for the smartphone (Figure 3a). In the test, we inserted the chip into the chip holder (Figure 3b,c). Then we centered the chip under the PDMS microlens/smarphone camera (Figure 3d) and adjusted the focus knob to acquire a clear image of the T-point and C-point in the CRP-Chip using the smartphone (Figure 3e,f). A blank area of the paper device was used as the background reference. 

### 2.7. Data Analysis

The images captured by the smartphone were first analyzed using ImageJ. After background subtraction, the grey intensity of the T-point was measured. Different concentration of CRP standard was used to generate the linear detection range of the CRP-Chip. The linear curve was used to calibrate the test image signal to the CRP concentration in samples. Statistical analyses were performed using Origin 8 (Origin Lab). The lower limit of detection (LOD) was calculated using the CRP standard calibration line (i.e., LOD = 3 × standard deviation of the regression line intercept/regression line slope). The Student’s *t*-test was used to compare the CRP test data of different samples. The difference between the two sets of data was considered statistically significant with *p* < 0.05. 

To enable rapid sample-to-result assay, we developed a smartphone app for image capture and data analysis of the CRP-Chip. The app was developed using the Android Studio Development Tool. An open source computer vision library, OpenCV, was integrated to the app to perform the image acquisition and processing. The data analysis procedure is similar to ImageJ (Figure 3g). Briefly, the captured image is displayed in the smartphone screen within the app. Then the T-points and a blank point as the background are selected. The app automatically calculates the grey intensity of the T-points and substrates the background. The CRP concentration is calculated by fitting the measured T-point signal to the pre-stored calibration curve. The three T-point measurements from a CRP-Chip are averaged and the final CRP concentration is displayed on the smartphone screen. All data were independently analyzed by the smartphone app and the results are consistent with the ImageJ analysis.

### 2.8. CRP Test Using Conventional ELISA Kit 

The CRP concentration in the CKD patients’ plasma and serum samples was measured by a commercial Human CRP ELISA Kit (DCRP00, R&D Systems Inc., Minneapolis, MN, USA) and a multi-plate reader at 450 nm (Synergy 4 HT). All the serum and plasma samples were initially diluted 100 times using the sample diluent buffer for the test according to the manufacturer’s recommendation. For the samples with high CRP concentration exceeding the ELISA kit’s detection range, the test was repeated with a 1000× dilution of the sample.

## 3. Results

### 3.1. Technical Validation and Characterization of the CRP-Chip

**Figure 4:**
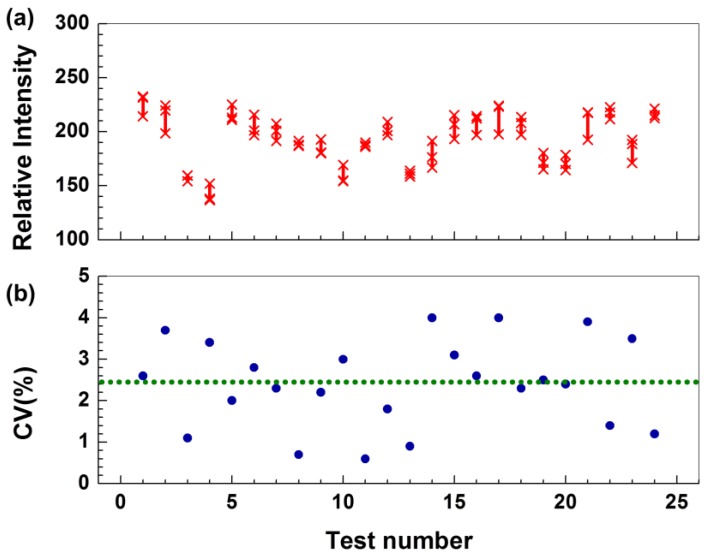
Reproducibility of the CRP test results using the CRP-Chip. (**a**) Relative T-point intensity of CRP test using CRP standard, plasma, and blood samples from 24 CRP-Chips. For each device, “x” indicates the measurements of the three T-points; (**b**) The corresponding coefficient of variation (CV) of the data in (**a**). The dotted line indicates the average CV for all the devices.

**Figure 5:**
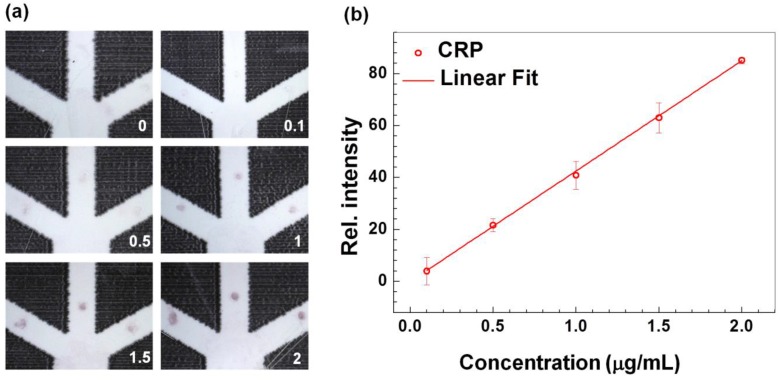
Technical validation and characterization of the CRP-Chip using the CRP standard. (**a**) Representative images of the T-point of different concentrations of CRP standard (0–2 μg/mL) using the CRP-Chip; (**b**) The calibration curve of the average T-point intensity against the CRP concentration. The error bars are the standard error of the mean (SEM). The solid line is the linear fit of the data (R^2^ = 0.9997; slope = 42.55).

To validate the CRP-Chip for effective detection of CRP from different samples and to characterize its test reproducibility, we first tested the CRP level in human CRP standards (diluted in PBS) and plasma or whole blood samples from healthy subjects. A single CRP-Chip could perform the CRP test in triplicate (i.e., three parallel test channels in a single chip) within 15 min for the total material cost of less than 50 US cents. Measurements of a given sample from the three test channels of a CRP-Chip were consistent with a 2.4% coefficient of variation (CV) (Figure 4). Using different concentrations of the human CRP standard, we determined that the general linear detection range of CRP using the CRP-Chip was up to 2 μg/mL (Figure 5). The lower limit of detection (LOD) of the CRP-Chip was determined as 54 ng/mL using the CRP standard calibration line. The test results were comparable between a freshly prepared CRP-Chip and the CRP-Chip stored at 4 °C for several days.

**Figure 6:**
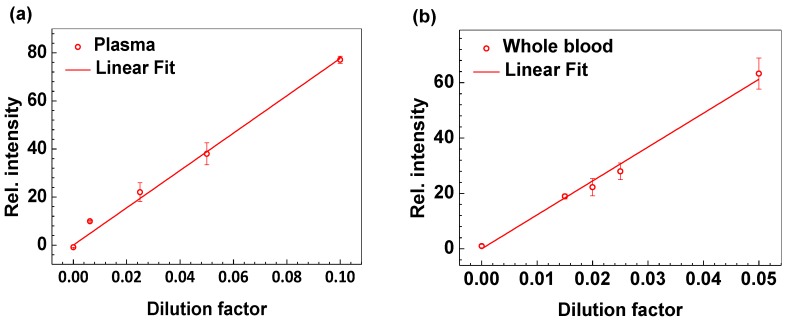
CRP test of plasma or whole blood from healthy donors using the CRP-Chip. The CRP-Chip test results linearly correlate with the sample dilution factor for plasma (**a**) (linear fit yields R^2^ = 0.994; slope = 776.76) and whole blood (**b**) (linear fit yields R^2^ = 0.996; slope = 1224.46). The samples were diluted in PBS. The dilution factor is calculated as the fraction of the diluted sample concentration relative to the undiluted sample. The error bars are the standard error of the mean (SEM).

Using the calibration curve from the CRP standard, we measured CRP from healthy blood donors’ isolated plasma and whole blood, and compared the results to the gold-standard ELISA. The CRP test results correlated very tightly with the dilution factor of the plasma or whole blood sample therefore allowing expanded detection range (Figure 6). The CRP test directly using whole blood was highly concordant with measurements in plasma, demonstrating that the CRP-Chip is capable of effectively testing whole blood using the chip’s integrated plasma separation module, eliminating the need for a separate plasma separation step.

### 3.2. Validation of the CRP-Chip for Testing Clinical CKD Patient Samples

**Figure 7:**
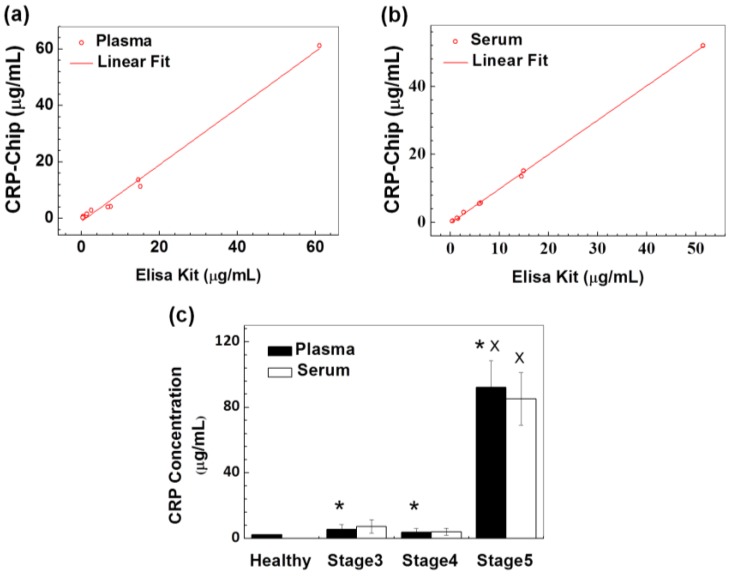
Validation of the CRP-Chip for CRP test of clinical samples from CKD patients. CRP test results using the CRP-Chip and conventional well-plate-based ELISA kit using CKD patients’ plasma samples (**a**) (linear fit yields R^2^ = 0.991; slope = 1.001) and serum sample (**b**) (linear fit yields R^2^ = 0.999; slope = 1.011) were compared; (**c**) Comparison of the CRP test results using the CRP-Chip for the total 17 plasma and serum samples from stage 3–5 CKD patients and from 10 healthy blood donors. * indicates *p* < 0.05 comparing to the healthy control (plasma only); x indicates *p* < 0.05 compared to stage 3 and stage 4 CKD patients (both plasma and serum).

To evaluate the CRP-Chip with clinical CKD patient samples, we tested the CRP level of 10 CKD patients’ plasma and serum samples, which were randomly selected from an established patient cohort (stages 3–5 of CKD). The disease stage of these 10 patients determined by the traditional eGFR-based method (Table 1) was not communicated to the researcher, who was doing the CRP test until the test was completed. The CRP test results using the CRP-Chip were in agreement with the traditional well-plate-based ELISA (Table 1; Figure 7a,b). The coefficient of determination (R^2^) of the CRP measurements between the CRP-Chip and ELISA kit is 0.996 for plasma samples and 0.999 for serum samples. Furthermore, the CRP level of the CKD patients measured by the CRP-Chip was generally higher than the healthy subjects. Among the CKD patients, the CRP level was significantly elevated in the stage 5 patient than other stage 3 and stage 4 patients (Table 1). Because the initial test only included one stage 5 CKD sample, we further tested a total eight stage 5 CKD samples from the same patient cohort using the CRP-Chip (Table 2). The test result confirmed the significantly higher CRP level in both plasma and serum of stage 5 CKD patients than stage 3 and stage 4 CKD patients (Figure 7c). This result is consistent with the known clinical correlation between blood CRP level and CKD stage.

## 4. Discussion and Conclusions

Diseases such as chronic cardiovascular and kidney disease are a growing epidemic worldwide, and are associated with catastrophic adverse clinical outcomes and costs [31,32]. Importantly, the growth in chronic diseases is often highest in areas of the world where healthcare access is limited. Effective intervention to prevent downstream complications such as death or organ failure requires early and accurate disease diagnosis and risk prediction [33,34,35]. Although instruments for predicting clinical risk are available, they remain imperfect [36,37,38]. New approaches based on protein CKD biomarkers can improve risk prediction and are thus effective targeting of therapy. However, the effective translation of novel biomarkers to clinical application is limited by the high cost and the high requirement of specialized facilities and skills for performing the assay. Our prototype CRP-Chip demonstrates the feasibility of effectively addressing the challenges of cost-factor and test speed for protein biomarker detection. Importantly, our CRP-Chip offers a PoC platform for testing different protein biomarkers individually or in combination. The wax printing method to define microfluidic channels in paper materials enables efficient prototyping of device designs and larger scale device manufacturing.

Microfluidics-based immunoassays [39,40,41] have important advantages over traditional assays including enhanced efficiency of chemical reactions and reduced reagent consumption. However, existing microfluidic immunoassays still require a specialized reading instrument. In addition, although some developments have shown promise in automating the operation of microfluidic immunoassays [42,43] and commercial products have been developed, fully automated on-chip immunoassays in a compact and portable format suitable for inexpensive PoC tests are not available. This developed CRP-Chip meets the requirement for PoC CKD diagnosis tool in rapid and semi-automated sample-to-result test, self-powered fluidic transport, low-cost, portable test signal reading, and analysis. The smartphone app can be further developed to enable a fully-automated sample-to-result test by incorporating the automatic selection function for data analysis. The LOD of the CRP-Chip is comparable to that of conventional LFA in the ng/mL range [44,45,46,47]. Collectively, this CRP-Chip represents a new application of the fast growing MS^2^ technology for potential PoC CKD diagnosis. However, the detection limit and sensitivity of the current CRP-Chip is a significant limitation comparing to more sensitive and accurate methods such as ELISA (e.g., >60 times higher for LOD; high detection sensitivity but at low resolution comparing to a commercial ELISA kit). On the other hand, the CRP-Chip has a good linear range comparing to ELISA. Thus, the CRP-Chip is limited to the protein biomarkers that fit to the detection specifications of the chip. 

It is worth mentioning that the specific CKD cohort in this study only includes stage 3–5 CKD patients. We found the CRP level of these advanced stage CKD samples is significantly higher than the healthy controls. Thus, this developed method has important clinical and research implications. Future development of this method to enable combinatorial multi-marker detection has the potential to improve CKD diagnosis. In addition, future development of the CRP-Chip by incorporating more sensitive detection technologies such as spectroscopy or new electrical or electrochemical sensors to the μPAD will permit applications of the device to measurement of other important biomarkers. The availability of fast, easy, and cheap biomarker measurement platform has the potential to accelerate clinical validation and translation of biomarkers into effective risk prediction tools. Ultimately, creation of an inexpensive multiplex CKD-Chip or CVD-Chip offering simultaneous measurement of multiple biomarkers relevant to each chronic disease will help effective disease screening, risk stratification, and targeted interventions leading to improved outcomes. 

## Figures and Tables

**Table 1:** Blind CRP test of 10 blood plasma and serum samples from CKD patients by traditional ELISA kit and the CRP-Chip. Blind CRP test of 10 blood plasma and serum samples from CKD patients by traditional ELISA kit and the CRP-Chip [CRP concentration is expressed in μg/mL].

	1	2	3	4	5	6	7	8	9	10
**Plasma (ELISA kit)**	15.1	1.24	6.8	0.45	14.6	2.52	7.51	1.39	0.32	**61.1**
**Plasma (CKD-Chip)**	11.3	0.92	4.10	0.65	13.6	2.90	4.13	1.60	0.28	**61.2**
**Serum (ELISA kit)**	14.9	1.56	5.9	0.41	14.5	2.73	6.15	1.35	0.33	**51.5**
**Serum (CKD-Chip)**	15.18	1.14	5.51	0.40	13.53	2.96	5.75	1.25	0.35	**52**
**CKD Stage**	3	3	3	4	4	4	4	4	4	**5**

**Table 2:** Clinical descriptors of 17 CKD patients tested in this study.

	Gender	Fasting	Dialysis	CKD stage	eGFR*	uACR#
1	F	N	Y	STAGE 3	33	60.7
2	F	Y	N	STAGE 3	40	2.3
3	M	N	N	STAGE 3	36	4.1
4	F	N	N	STAGE 4	22	452.3
5	F	N	Y	STAGE 4	23	0.8
6	M	N	N	STAGE 4	27	27
7	F	Y	N	STAGE 4	26	0.6
8	M	N	N	STAGE 4	24	11.2
9	F	N	Y	STAGE 4	22	334.5
**10**	**M**	**N**	**Y**	**STAGE 5**	**13**	**47.9**
**11**	**F**	**Y**	**Y**	**STAGE 5**	**8**	**502.7**
**12**	**M**	**N**	**Y**	**STAGE 5**	**11**	**302.2**
**13**	**F**	**N**	**Y**	**STAGE 5**	**11**	**698.4**
**14**	**M**	**Y**	**N**	**STAGE 5**	**9**	**144.8**
**15**	**M**	**N**	**Y**	**STAGE 5**	**12**	**433**
**16**	**M**	**Y**	**Y**	**STAGE 5**	**11**	**>656.7**
**17**	**F**	**Y**	**N**	**STAGE 5**	**10**	**156.9**
